# Influences of altering footstrike pattern and cadence on lower extremity joint coordination and variability among runners with patellofemoral pain

**DOI:** 10.1371/journal.pone.0280477

**Published:** 2023-01-23

**Authors:** Yue Liu, Yujie Qi, Yanliqing Song, Li Feng, Lin Wang

**Affiliations:** 1 Key Laboratory of Exercise and Health Sciences (Shanghai University of Sport), Ministry of Education, Shanghai, China; 2 School of Kinesiology, Shanghai University of Sport, Shanghai, China; 3 Nan Xiang Community Healthcare Center, Shanghai, China; 4 College of Sports, Nanjing Tech University, Nanjing, China; 5 Affiliated Sport Polytechnic, Shanghai University of Sport, Shanghai, China; 6 Department of Sports Rehabilitation, Shanghai University of Sport, Shanghai, China; Ningbo University, CHINA

## Abstract

**Background:**

Patellofemoral pain (PFP) is a common overuse injury among runners. It is not only a hindrance to the runner’s training, but also to the runner’s quality of life. PFP runners may strategize different running strategies to reduce patellofemoral joint stress, release pain, and improve function.

**Purpose:**

This study aimed to determine the changes in joint coordination and variability under combinations of foot strike pattern and cadence for runners with patellofemoral pain.

**Methods:**

Twenty male runners with PFP performed six running strategies which were two strike patterns named forefoot (FFS) and rearfoot (RFS) accompanied by three running cadences named slow10%, normal, and fast10%. A modified vector coding technique and circular statistics were respectively used to identify the coordination pattern and variability between hip sagittal-knee frontal (HsKf), hip sagittal-knee sagittal (HsKs) and knee transverse-ankle frontal (KtAf) during stance phase. Coordination patterns which were conformed with anatomical motion pattern was classified as mechanically sound, and the distribution frequency of each coordination pattern was quantified.

**Results:**

Switching to FFS, the HsKf couples (p < 0.001, ES = 1.34) and the HsKs couples (p = 0.001, ES = 0.82) displayed significantly greater frequency in mechanically unsound coordination pattern during the initial stance phase. The effect of increasing running cadence on RFS displayed significantly greater frequency in mechanically unsound hip dominancy (p = 0.042, ES = 0.65) and knee dominancy (p = 0.05, ES = 0.70) coordination patterns for HsKf couples as well as for HsKs couples (p = 0.023, ES = 0.86) during the initial stance phase. Combined with FFS and fast10% cadence, HsKs couples showed more hip-dominated mechanical sound coordination pattern (p = 0.002, ES = 1.25). Further, altering footstrike pattern and cadence failed to change the coordination variability.

**Conclusions:**

Changing running cadence (± 10%) combined with transfer strike pattern from RFS to FFS could not increase the distribution frequency in mechanically sound coordination patterns and change coordination variability for PFP runners.

## Introduction

Patellofemoral pain (PFP) is a common lower limb musculoskeletal overuse injury among runners. The prevalence of PFP varies by population and may be as high as 20%–25% in active populations [1, 2]. PFP is characterized by diffuse anterior knee pain and usually associated with activities, such as walking, running, squatting, and stair ascent and descent [3]. It is often persistent and could continue for many years and eventually develop into irreversible patellofemoral osteoarthritis, which in turn negatively influences the quality of life [4].

Although the exact biomechanical pathogenesis of PFP is still not well understood, the possible mechanism underlying the development of PFP is abnormal loading of patellofemoral joint and may be influenced by kinematic factors during gait [4]. Greater knee valgus in the frontal plane during running of individuals with PFP [5, 6] contributes to increased lateral components of patellofemoral joint loading and to pain [7]. Despite claims that abnormal knee flexion is linked to increased patellofemoral joint stress, existing literature remains controversial. Most studies have reported that runners with PFP have increased peak knee flexion compared with asymptomatic runners [8], but other works failed to prove it [6]. Similarly, knee motion in the transverse plane is not consistent across studies; several existing studies showed that knee internal rotation increased in runners with PFP compared with healthy runners [9], whereas others did not [10]. In addition, rearfoot valgus may be one of the biomechanical risk factors causing PFP [11]. The joint motion changes in patients with PFP in different motion planes remain controversial. Runners with PFP may strategize their running strategies to reduce patellofemoral joint stress, release pain, and improve function. Compared with the preferred running cadence, the cumulative patellofemoral load impulse and peak knee flexion decreased by 5.5% and 3.3 degree (7.0%), respectively, in stance phase at 110% step cadence among healthy individuals [12]. Furthermore, Bonacci found that increased running cadence or minimalist footwear have greater variability, which may help distribute loads for patients with PFP [13]. Transitioning from a rearfoot to forefoot strike pattern increased the Lower Extremity Function Scale score and led to greater gastrocnemius and rectus femoris pre-activation during the late swing phase, thereby benefitting runners with PFP [14, 15]. However, in these studies, the effect of a single running strategy change on patients with PFP was compared; research evaluated discrete variables from the dynamic perspective of force and moment.

From the point of view of dynamical system theory, human movement requires the complex organization of multiple degrees of freedom to form coordinated action for coping with external interference or different task conditions. Vector coding technique is a widely used method to quantitatively analyze coordinated action from joint coordination and coordination variability to provide insights into spatial variation between joints with time series and organized flexibility between joints during different motions [16]. The spatial variation information between joints is represented by coordination pattern, which may be dysfunctional if the movement does not conform to anatomical linkages between joints (mechanically unsound) [17]. Mechanically unsound coordination relationship between knee and ankle can increase the risk of joint exposure to abnormal stress and could be related to PFP [18]. In addition, based on variability–overuse injury hypothesis, a lower coordination variability may lead to repeated loads on specific tissues and is more likely to cause overuse injury [19]. Heiderscheit [20] found no differences in coordination variability between PFP and healthy runners when calculating the average across the entire running gait cycle at a self-selected running speed. Conversely, Cunningham indicated that greater coordination variability may be a characteristic of female runners with PFP [19]. Hence, a dynamic system approach is needed to comprehensively determine the coordination relationship and variability between the joints of lower limbs in different planes while running in different combinations of strategies among runners with PFP.

This study aimed to determine the joint coordinative patterns and coordination variability when changing the running cadence and strike pattern in runners with PFP. The authors hypothesized that runners with PFP could display more mechanically sound coordinative patterns and a greater CAV with increasing running cadence and altered strike pattern to forefoot strike pattern.

## Methods

### Participants

Twenty male runners (age, 22.6 ± 2.6 years; body height, 171.8 ± 5.4 cm; body weight, 72.1 ± 9.1 kg) were recruited from the university by using flyers and posters. The sample size was calculated based on the lower extremity coordination variability observed in previous work indicated that effect size of 0.37, power of 0.8, and a significance level of 0.05, 12 participants were required for adequate statistical power [13]. The inclusion criteria were as follows: 1) aged 18–45 years; 2) regular exercise habit runners or athletes; 3) habitual rearfoot strikers; 4) self-report of knee pain during and/or after running training in the last 3 months, unrelated to any traumatic event, and worst knee pain experienced in the previous week of 3–7 out of 10 [using a 10 cm Visual Analogue Scale (VAS, ‘0’ indicating no pain and ‘10’ indicating extremely intense pain)]; 5) PFP symptom may occur in at least two activities other than running: squatting, walking up or/and down stairs, kneeling, jumping and prolonged sitting [21]. Participants were excluded if they had: 1) a history of knee surgery; 2) inability to perform a lunge/squat, patellar tendinitis or joint effusion or other neurological disorders that affect normal gait. A licensed physical therapist screened all potential participants for inclusion and exclusion [14].

All participants signed a written informed consent before participating and the study procedure was approved by the Human Ethics Committee of Shanghai University of Sport (ID: 102772020‬RT002).‬‬‬‬‬‬‬‬‬‬‬‬‬‬‬‬‬‬‬‬‬‬‬‬‬‬‬‬

### Instrumentation

Markers’ trajectory was recorded using a three-dimensional motion capture system with 8-camera (Vicon T40, Oxford Metrics, Oxford, UK) sampling at 100 Hz [22] synchronously with ground reaction forces from two 1.75 × 0.5 m force platforms at 1000 Hz (Bertec instrumented treadmill, Columbus, OH, USA). Twenty-eight 10-mm retroreflective markers were attached to the prescribed anatomical locations as per plug-in gait lower body marker setup (Plug-In Gait Reference Guide, 2018). Following the application of markers, static calibration data were collected for each participant. A notable detail that to minimise the confounding factors relating to footwear, all participants wore standard shoes and socks with the same brand and material.

### Protocol

All tests were conducted in an indoor laboratory and the participants were instructed to complete 5 minutes of warm up on the treadmill at preferred speed for a 5km running. For the last 2 minutes of warm up, the running speed was recorded and the number of foot strikes over a 30-s period was counted and multiplied by 2 as the standard speed and normal cadence of gait retraining [23]. Each participant was familiarised with six different running strategies test under the guidance of a supervised experimenter, each of which lasted about 5 minutes with rearfoot strike pattern (RFS) and forefoot strike pattern (FFS) and three cadences (slow = normal cadences −10%, normal, and fast = normal cadences +10%). A metronome was used to set the target running cadence and verbal feedback was provided by the physiotherapist to adjust cadence in accordance with this metronome. The foot strike pattern was confirmed through strike index. If runners first touched the ground with the posterior 1/3 of the foot length would be classified as RFS. If the anterior 1/3 of the foot length touched the surface would be named as FFS [24]. After a full rest, the participants were asked to complete six different running patterns for 30s at slow, normal and fast cadences using RFS and FFS, respectively, on treadmill in random order. Five successful gait cycle for each pattern were selected, and the stance phase of each gait cycle were used in data analysis [25]. The gait cycle was deemed successful if participants ran within the target cadence and correct landing pattern.

### Date processing

Joint kinematics were extracted for the ankle, knee and hip. Kinematic data were filtered with a low-pass fourth-order Butterworth filter at a cut-off frequency of 10 Hz [26]. Ankle, knee and hip joint rotations were calculated on the basis of Euler rotation methods (X: flexion/extension, Y: adduction/abduction, Z: internal/external rotation). Vertical ground reaction forces were sample at 1000Hz with a threshold of 20N to detect gait cycle events of the stance phase [27]. Stance phase was time normalised from initial contact (0%) to toe off (100%). Stance was divided into three portions: initial stance (0%–33%), mid stance (34%–66%) and late stance (67%–100%) [18]. Coupling angle (γ) and coupling angle variability (CAV) of hip sagittal versus knee frontal (HsKf), hip sagittal versus knee sagittal (HsKs) and knee transverse versus ankle frontal (KtAf) couples were calculated using circular statistics known as modified vector coding technique [28]. Modified vector coding analysis was performed using a custom-written MatLab code (MatLab 2016a, MathWarks, Inc., Natick, MA).

The coupling angles were calculated on the basis of proximal joint angles (θ_*P*_) and distal joint angles (θ_*D*_) by using the following formula to present values between 0° and 360°:

$$\gamma_{j,i}=\tan^{-1}(\frac{\theta_{D(i+1)}-\theta_{Di}}{\theta_{P(i+1)}-\theta_{Pi}})∙\frac{180}{\pi }$$ (1)

where *i* is a percent running cycle of the *j*th trial. And as suggested by Needham, the coupling angles were classified into eight coordination patterns on the basis of coupling angle magnitude [28] (Table 1). The distribution frequency of eight coordination patterns were quantified to reveal the dominant coordination pattern for each coupling pair during the stance phase. Coordination patterns were qualitatively interpreted as mechanically sound or unsound according to the anatomical motion pattern and polarity defined by the right hand rule. Mechanically sound coordination patterns indicated that hip, knee and ankle move as they are anatomically linked [17]. Therefore, during the running stance phase, mechanically sound motion included:

hip extension (EXT)–knee eversion (EV), hip flexion (FLX)—knee inversion (INV);

hip extension (EXT)—knee flexion (FLX), hip flexion (FLX)—knee extension (EXT);

knee internal rotation (IR)—ankle eversion (EV), knee external rotation (ER)—ankle inversion (INV).

Any opposite coordinative pattern was defined as mechanically unsound.

CAV was calculated in accordance with Eqs (2–5) as follows:

$${\bar{x}}_{i}=\frac{1}{n}\sum_{i=1}^{n}\cos\;\gamma_{i}$$ (2)

$${\bar{y}}_{i}=\frac{1}{n}\sum_{i=1}^{n}\sin\;\gamma_{i}$$ (3)

$${\bar{r}}_{i}=\sqrt{{{\bar{x}}_{i}}^{2}+{{\bar{y}}_{i}}^{2}}$$ (4)

$${CAV}_{i}=\sqrt{2(1-{\bar{r}}_{i})}∙\frac{180}{\pi }$$ (5)

**Table 1:** Classification of coordination patterns.

Coordination pattern	Coupling angle (γ)	HsKf	HsKs	KtAf
Inphase proximal dominance	0°≤ γ < 45°	*		
	180°≤ γ < 225°	*		
Inphase distal dominance	45°≤ γ < 90°	*		
	225°≤ γ < 270°	*		
Antiphase distal dominance	90°≤ γ < 135°		*	*
	270°≤ γ < 315°		*	*
Antiphase proximal dominance	135°≤ γ < 180°		*	*
	315°≤ γ < 360°		*	*

The abbreviations used are as follows: D: distal joint, P: proximal joint, *indicates that motion is mechanically sound.

### Statistical analysis

SPSS version 26.0 (SPSS Inc., Chicago, USA) was used for statistical analysis. Shapiro–Wilk tests revealed a non-normal distribution of the coordination patterns and CAV. Therefore, Wilcoxon rank-sum tests (α < 0.05) were conducted to identify differences amongst three running cadences in the distribution frequencies of coupling angles and CAV during stance subphase at two strike patterns. Friedman tests (α < 0.05) were conducted to identify differences between two strike patterns in the distribution frequencies of coupling angles and CAV during stance subphase at three running cadences.

## Results

### Coordination patterns

**Fig 1:**
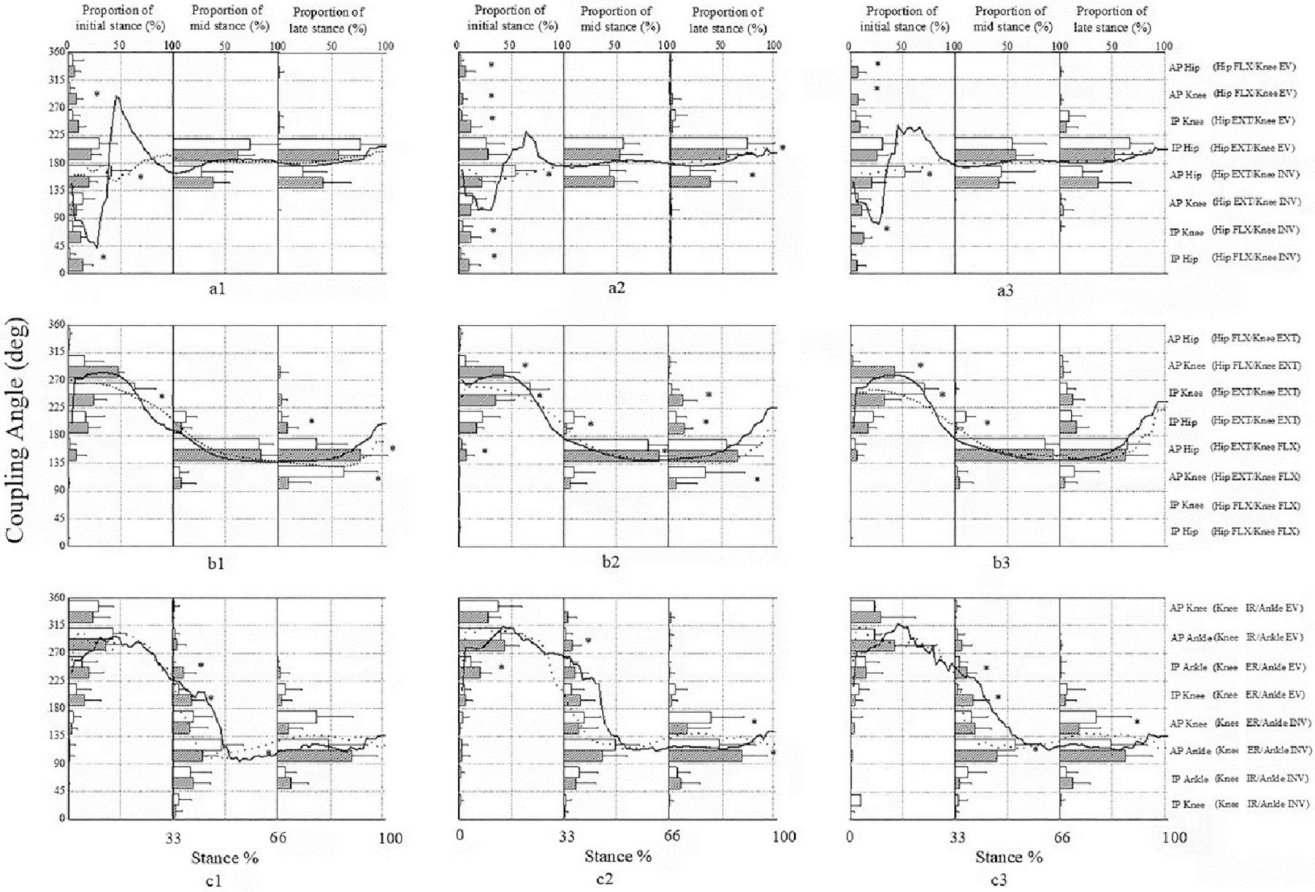
Mean coupling angle in RFS and FFS conditions. Mean coupling angle for all subjects across stance phase for hip sagittal versus knee frontal (a), hip sagittal versus knee sagittal (b) and knee transverse versus ankle frontal (c) couples in RFS (solid line) and FFS (dash line) during slow10% (left column), normal (middle column) and fast10% (right column) running cadence. Each figure is divided into three parts, which represent the distribution proportion of RFS (slash) and FFS (white) in each coordination pattern during substance phases. * indicates a significant difference between groups (p < 0.05).

**Fig 2:**
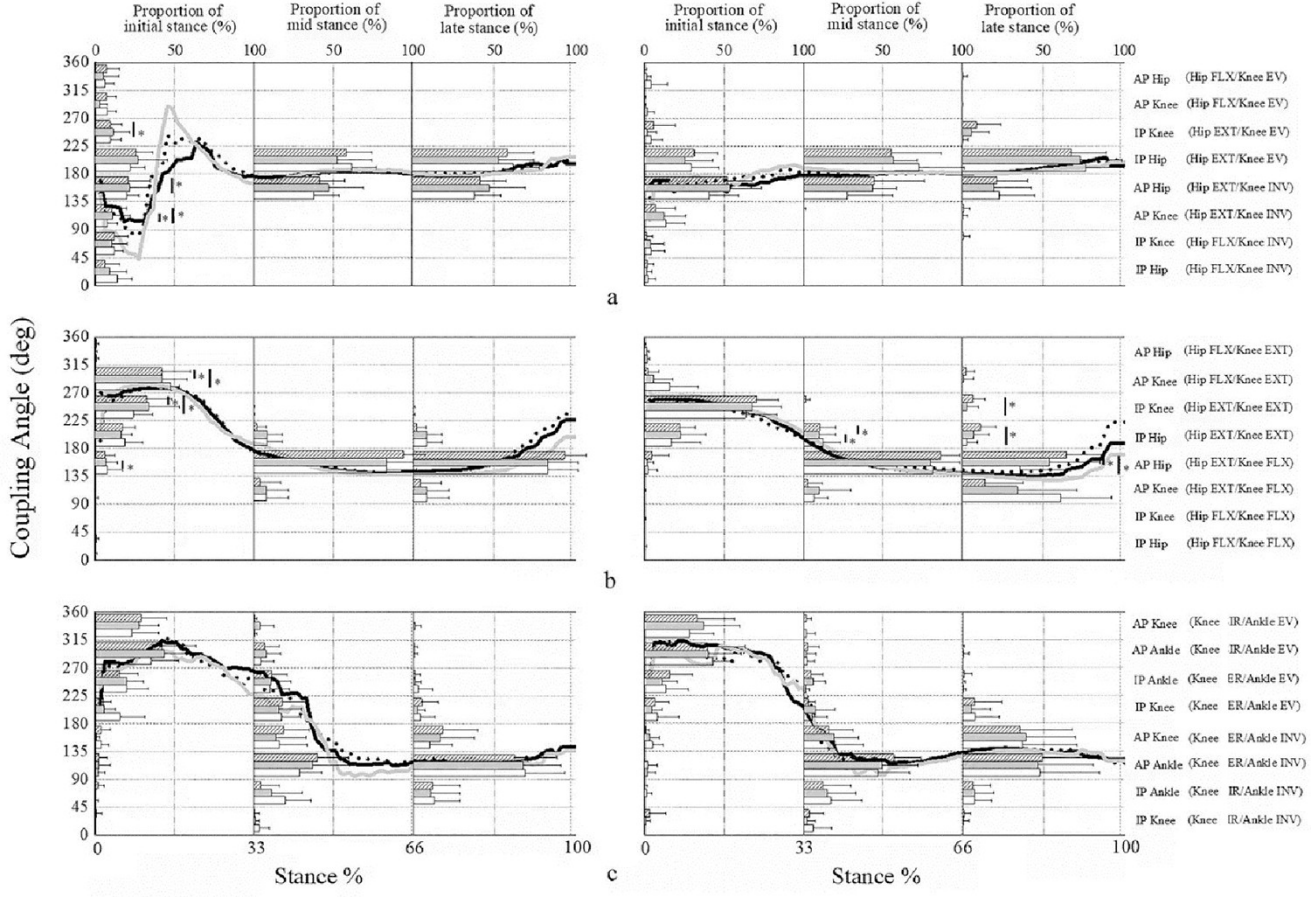
Mean coupling angle during slow 10%, normal and fast 10% running cadence. Mean coupling angle for all subjects across stance phase for hip sagittal versus knee frontal (a), hip sagittal versus knee sagittal (b) and knee transverse versus ankle frontal (c) couples in RFS (left column) and FFS (right column) during slow10% (grey line), normal (dash line) and fast10% (black line) running cadence. Each figure is divided into three parts, which represent the distribution proportion of slow10% (white), normal (grey) and fast10% (slash) in each coordination pattern during initial (left column), mid (middle column) and late (left column) stance phases. * indicates a significant difference between groups (p < 0.05).

For HsKf couples, coordination patterns were concentrated in in-phase hip EXT-knee EV (mechanically sound) and anti-phase hip EXT-knee INV (mechanically unsound) for FFS and RFS. Specifically, FFS displayed more frequent anti-phase hip EXT-knee EV (mechanically unsound) coordination pattern than RFS under slow10% (p = 0.005, ES = 1.61), normal (p < 0.001, ES = 1.34), and fast10% (p = 0.001, ES = 0.99) running cadence conditions (Fig 1) during the initial stance phase. During the mid and late stance phases, RFS showed more anti-phase hip EXT-knee INV (mechanically unsound) (p = 0.008, ES = 0.67) than FFS. In addition, running cadence exerted no significant effect on the distribution of coordination pattern among FFS runners. For RFS, the influence of cadence on the distribution was detected in anti-phase (mechanically unsound) hip dominancy (p = 0.042, ES = 0.65) and knee dominancy (p = 0.05, ES = 0.70) coordination patterns during the initial stance phase (Fig 2).

For HsKs couples, RFS displayed a large proportional anti-phase hip FLX-knee EXT (mechanically sound), while FFS displayed a large proportional in-phase hip EXT-knee EXT (mechanically unsound) under slow10% (p = 0.003, ES = 1.01), normal (p = 0.001, ES = 0.82), and fast10% (p = 0.001, ES = 0.75) running cadence conditions during the initial stance phase (Fig 1). Hip dominance anti-phase hip EXT-knee FLX (mechanically sound) was the most important coordination pattern for two strike patterns during the mid and late stance phases. However, as the running cadence increased, the coordination pattern gradually transferred from knee dominancy to hip dominancy during the late stance phase for FFS.

For KtAf couples, FFS showed a large proportion in ankle dominance anti-phase knee dominancy knee ER-ankle INV (mechanically sound) under slow (p = 0.012, ES = 0.75) and fast10% running cadence (p = 0.009, ES = 0.77) conditions in the mid stance phase. FFS also showed a large proportion in knee dominance anti-phase knee ER-ankle INV (mechanically sound) under normal (p = 0.008, ES = 0.92) and fast10% running cadence (p = 0.01, ES = 0.79) conditions in the late stance phase (Fig 1). No effect was observed on the distribution of coordination patterns under the three running cadence conditions for RFS and transfer to FFS (Fig 2).

### Coordination variability

Modified vector coding analysis revealed no differences in coordination variability across three portions of stance phases in HsKf, HsKs and KtAf couples under six conditions (Table 2).

**Table 2:** M (P25 and P75) for coordination variability of selected couples at six running conditions during three stance subphases.

		HsKf		HsKs		KtAf	
		RFS	FFS	RFS	FFS	RFS	FFS
Initial stance	Slow	51.31(50.43,57.64)	41.10(40.25,46.74)	52.79(51.40,58.19)	40.80(39.32,45.77)	48.29(47.03,55.03)	39.04(38.57,42.02)
	Normal	44.49(44.30,48.94)	41.66(41.44,45.53)	46.82(46.36,49.45)	39.54(39.06,43.47)	44.30(44.20,47.91)	39.60(38.48,42.37)
	Fast	44.10(43.29,48.54)	35.90(35.39,39.76)	47.30(46.98,49.76)	37.80(37.78,41.49)	46.39(45.11,49.70)	34.82(34.66,38.94)
Mid stance	Slow	51.85(51.01,57.00)	39.77(39.01,43.72)	51.04(49.86,58.75)	41.99(41.77,46.28)	51.12(50.36,55.40)	41.30(40.77,48.26)
	Normal	44.59(44.56,48.4)	38.53(37.71,42.16)	45.17(44.74,48.6)	41.91(41.52,44.79)	46.54(45.97,50.20)	40.45(40.00,43.90)
	Fast	46.32(44.77,50.47)	33.45(32.44,39.31)	45.23(44.80,48.19)	36.34(35.49,40.38)	44.42(43.93,48.53)	33.31(32.95,38.10)
Late stance	Slow	47.27(46.96,52.02)	40.31(39.5,44.95)	51.20(50.26,56.45)	39.52(38.21,44.22)	49.47(48.96,52.77)	42.46(41.85,46.80)
	Normal	45.14(44.87,47.98)	38.52(37.57,42.12)	45.38(45.14,48.94)	40.06(39.81,42.51)	44.94(44.35,47.63)	37.98(37.59,42.20)
	Fast	45.80(45.46,49.59)	35.99(35.15,39.24)	44.84(44.17,50.63)	33.79(33.18,37.93)	47.82(46.82,52.22)	33.46(33.37,37.70)

## Discussion and implications

This study aimed to determine whether alternating running cadence and strike patterns immediately affect joint coordination patterns and variability during the stance phase in runners with PFP. The results showed that the proportion of each coordination pattern throughout the running gait stance phase was changed in the six running strategies. However, changing strike pattern and running cadence did not show the same trend (mechanically sound/unsound) in the change in the distribution frequency of the coordination pattern, which contradicted our research hypothesis.

On the basis of the modified vector coding technique, the distribution frequency statistics indicated whether a coordination pattern performed a greater proportion of stance phase under different running strategies. For HsKf couples, the results highlighted the important role of the initial 1%–33% period of the stance phase among the six running strategies. Similarly, a previous research revealed that the most important kinematic change by transitioning from RFS to FFS was found in the knee frontal plane movement at initial contact in patients with PFP [14]. Switching to FFS and increasing the running cadence increased the distribution frequency in anti-phase hip EXT-knee INV (mechanically unsound) coordination pattern. This finding is similar to the results of previous research, revealing that the most important kinematic change in the gait retraining of patients with PFP was the change in knee frontal plane movement at initial contact [14]. Considering hip and knee motion during early stance, the knee continues inversion for a period of time and eversion, while the hip remains in extension. Thus, the mechanically unsound coordination pattern is the result of knee inversion after initial contact. The distribution frequency in the in-phase (mechanically sound) coordination pattern for HsKf couples was increased by altered strike pattern to FFS during the mid and late stance phases. From this point of view, we may not be able to simply explain which running cadence is better than the other. The frequency and patterns of couples may have a critical threshold [16]. However, whether the increase in running cadence has no effect on mechanically sound coordination patterns in either RFS or immediately switched to FFS remains unknown.

In this study, the anti-phase coordination pattern indicated mechanically sound motion between hip and knee in the sagittal plane during the stance phase. Proximal or distal dominancy stand for the joint rotating at a faster rate [29]. Under the condition of slow 10% and normal running cadence, simply switching the strike pattern to FFS will increase the distribution frequency in knee-dominated coordination pattern during the mid and late stance phases. As such, switching to FFS will lead to a faster rotating rate of knee flexion than hip extension, indicating that more load was transferred to the knee joint and less to the hip joint [26]. Notably, combining FFS and fast 10% cadence resulted in a change from knee dominancy to hip dominancy and tended to be RFS, a mechanically sound coordination pattern. Hence, hip dominancy coordination pattern may be indicative of another protective strategy in the PFP group in the propulsion phase. Overall, RFS or the combination of FFS and increased 10% cadence showed a greater distribution in hip dominancy coordination pattern, which may have implications for natural FFS runners with PFP. However, a healthy control group and natural FFS runners were not used for comparison, which is a limitation of the present research. Future research should focus on whether a relative angular velocity might produce potential information for the mechanism of runners with PFP.

From the anatomical point of view, knee external rotation-ankle inversion was defined as mechanically sound. No changes were found in the coordination patterns when slow10% or fast10% running cadence for RFS and transfer to FFS conditions. FFS performed a partial transformation from ankle dominancy to knee dominancy, while RFS remained in ankle dominancy during the last two stance phases. As previously discussed, knee dominancy coordination pattern would increase the demand on knee external rotation while simultaneously decreasing the demand on ankle inversion, thereby benefitting runners with PFP [26], despite that knee ER-ankle INV was the mechanically sound coordination pattern.

Inconsistent with the hypothesis, coordination variability did not appear to change with changing running cadence and strike pattern. Although no statistical difference was found in the variability of each condition, the variability tended to decrease with the acceleration of running cadence (Table 1). Our results did not corroborate the finding that 6 weeks of increased cadence gait retraining program increased the joint kinematic and kinetic variability [13]; notably, different statistical methods were used in our study. Care should be taken when using vector coding technique to determine the effect of long-term gait retraining from the perspective of variability.

Several limitations should be noted in this study. First, the treadmill condition could be considered a limitation. Treadmill was used to control the running speed to develop the relationship between gait retraining and joint coordination. Second, only the coordination patterns and variability of three couples were analyzed because they have strong kinematic coupling when running and they are related to PFP. Third, the lack of a health control group led to insufficient explanation on whether the coordination pattern was beneficial for PFP. The primary objective of the present study was to investigate whether changes in running cadence and strike pattern can alter the coordination pattern and variability of PFP. Additionally, the 20 participants included had VAS ranging from 3 to 7, which may affect the results. Future studies could increase the sample size and conduct subgroup analysis based on VAS value.

## Conclusions

Our findings suggest that immediately changing the running cadence (± 10%) and transferring strike pattern from RFS to FFS did not change the distribution frequency in mechanically sound coordination patterns and did not affect the coordination variability of runners with PFP. RFS exhibited more hip- and ankle-dominated mechanically sound coordination patterns when changing the running cadence in a short-term, which may be a protective strategy for natural RFS runners with PFP. Furthermore, additional studies are necessary to assess the long-term combination gait retraining effect on runners with PFP.
